# Supplementary material for: panX: pan-genome analysis and exploration
Source: Nucleic Acids Res. 2017 Oct 25;46(1):e5. doi: 10.1093/nar/gkx977 (PMC5758898; doi:10.1093/nar/gkx977)
Supplement: Supplementary Data [file gkx977_supp.zip › nar-02081-met-z-2017-File010.pdf]

panX: pan-genome analysis and exploration

Supplementary material

Wei Ding[1], Franz Baumdicker[2], Richard A. Neher,[1,3*]

[1]*Max Planck Institute for Developmental Biology,*

72076 Tübingen, Germany, [2] *Mathematisches Institut,*

Albert-Ludwigs University of Freiburg,

79104 Freiburg, Germany, [3] *Biozentrum,*

University of Basel, 4056 Basel, Switzerland

(Dated: September 27, 2017)

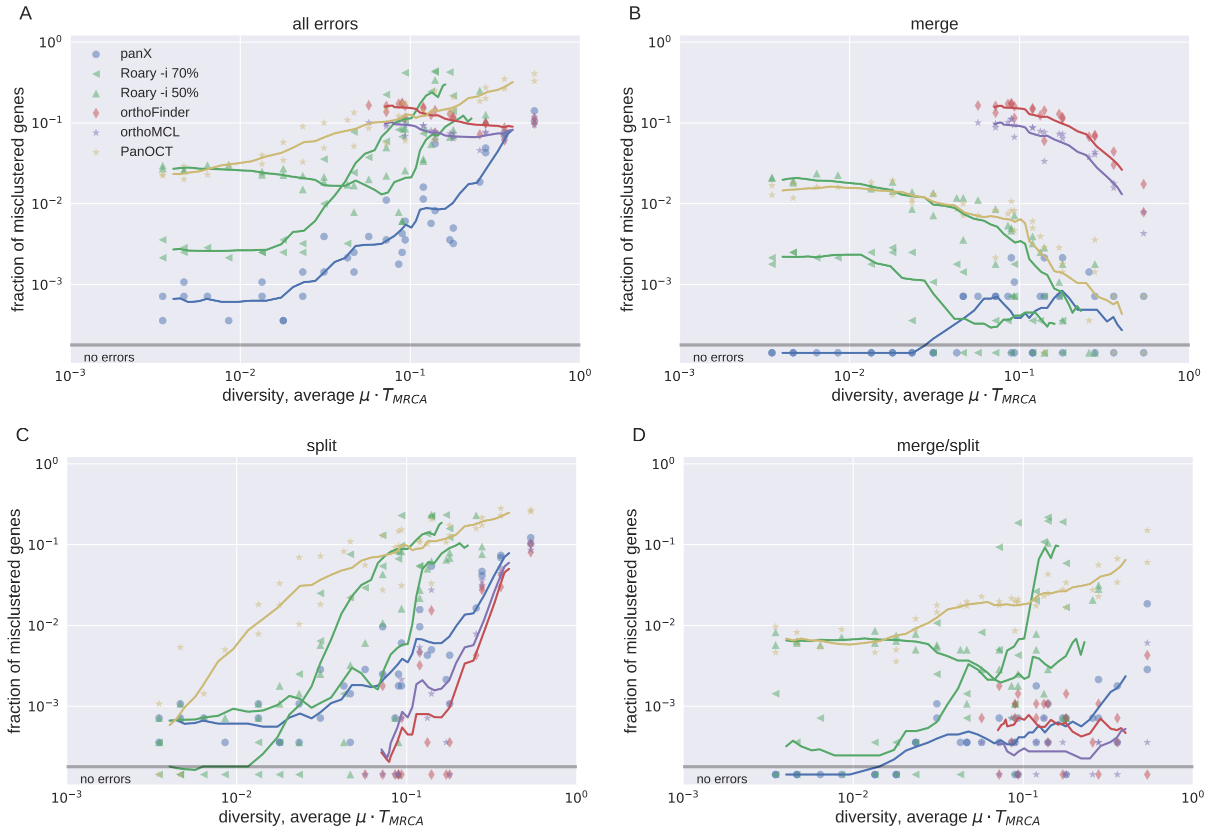

FIG. S1: Accuracy of clustering by types of clustering error and different tools for high gene conversion rater. Panel A shows the sum of all clustering errors for different pan-genome tools as a function of the pan-genome diversity. Panels B-D show the fraction of clusters that contain additional genes (merge errors), incomplete clusters (split errors), and clusters that miss genes and contain extra genes (merge/split), respectively.

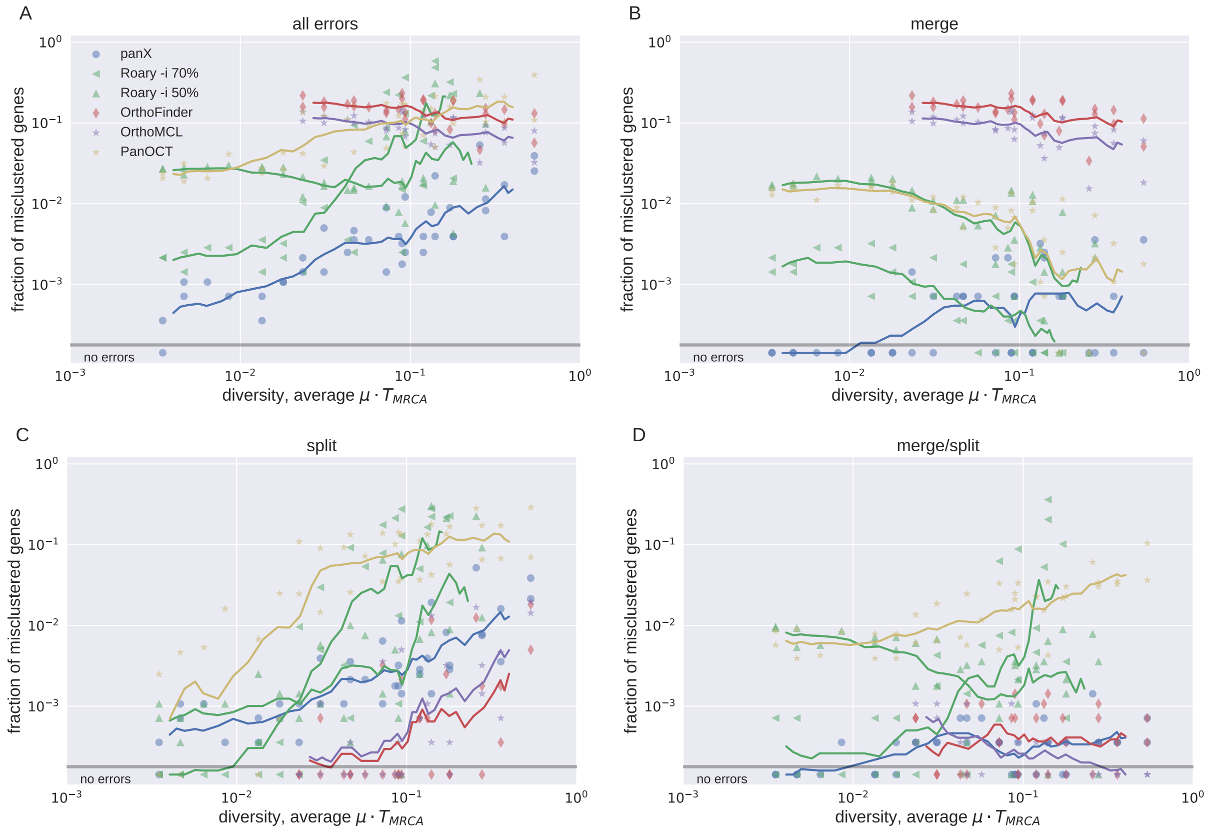

FIG. S2: Accuracy of clustering by types of clustering error and different tools for lowered gene conversion rate. Panel A shows the sum of all clustering errors for different pan-genome tools as a function of the pan-genome diversity. Panels B-D show the fraction of clusters that contain additional genes (merge errors), incomplete clusters (split errors), and clusters that miss genes and contain extra genes (merge/split), respectively.

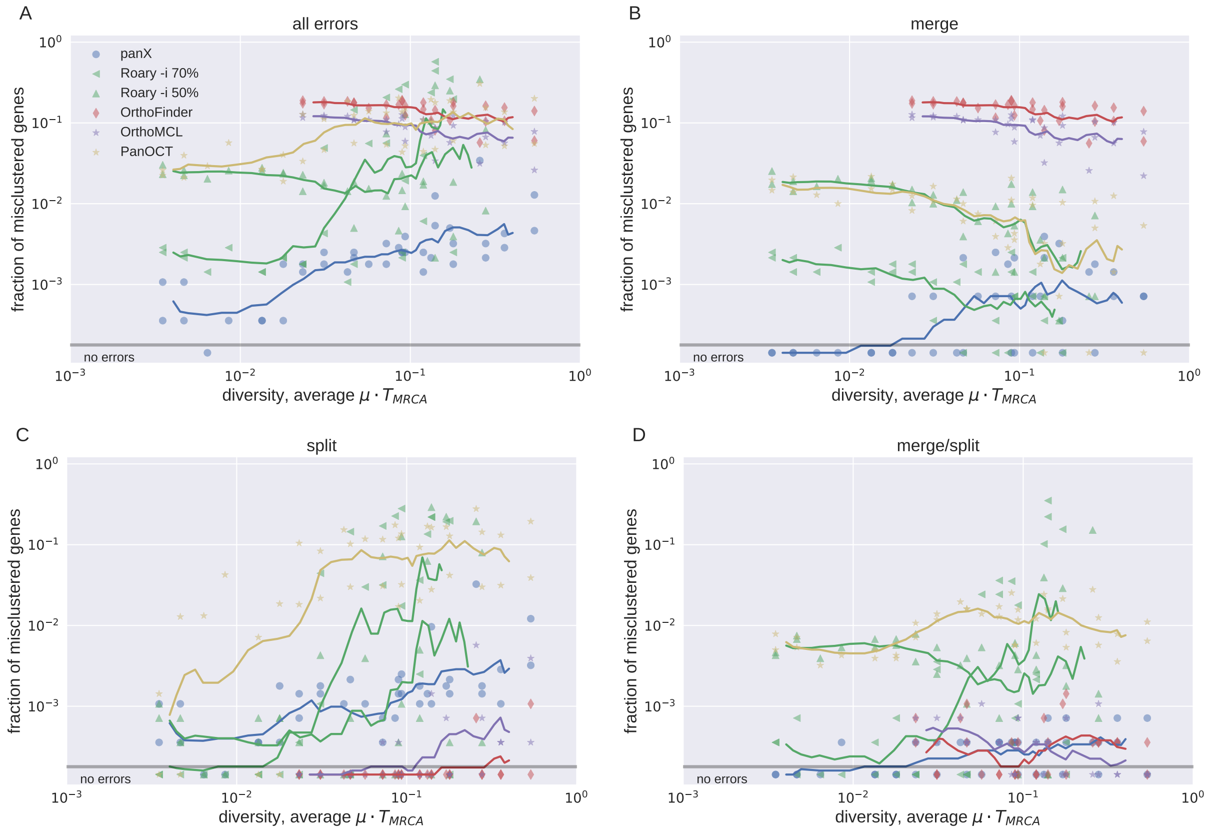

FIG. S3: **Accuracy of clustering by types of clustering error and different tools without gene conversion.** Panel A shows the sum of all clustering errors for different pan-genome tools as a function of the pan-genome diversity. Panels B-D show the fraction of clusters that contain additional genes (merge errors), incomplete clusters (split errors), and clusters that miss genes and contain extra genes (merge/split), respectively.

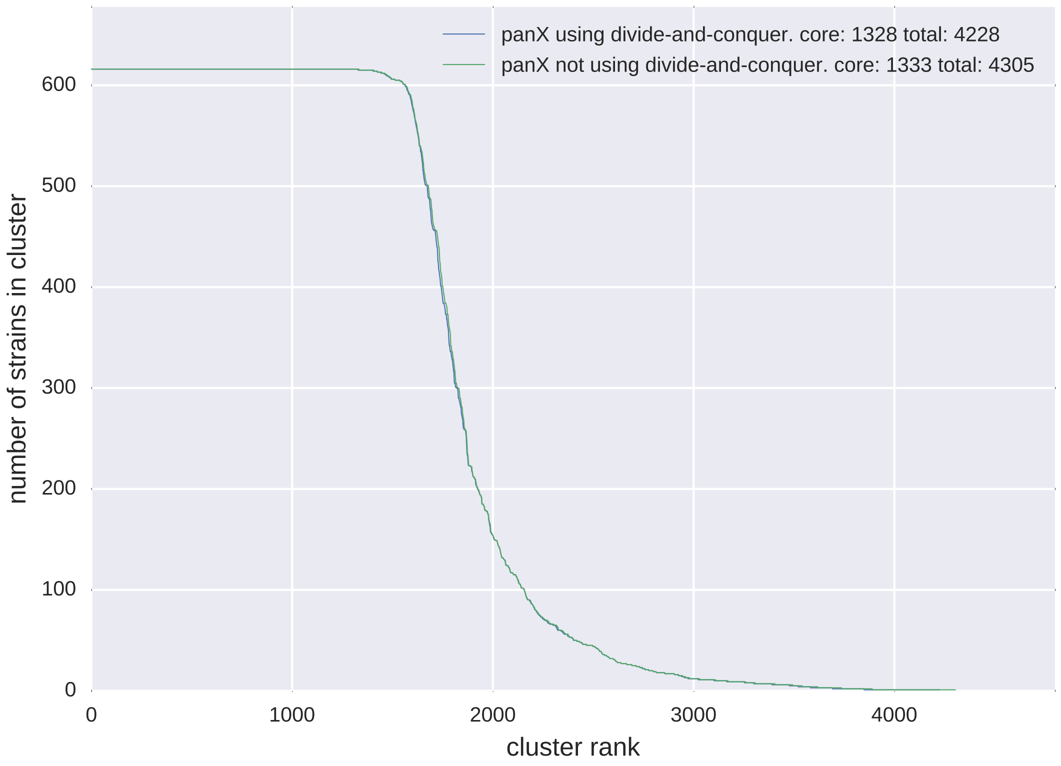

FIG. S4: The gene cluster size distributions inferred from the large dataset of 616 *S. pneumoniae* strains using panX with divide-and-conquer and panX without divide-and-conquer are very similar. The graph shows the inverse cumulative distributions. The numbers of core genes are almost identical (1328 vs 1333), while the clustering with the divide-and-conquer strategy infers a slightly smaller number of clusters (4228 vs. 4305).

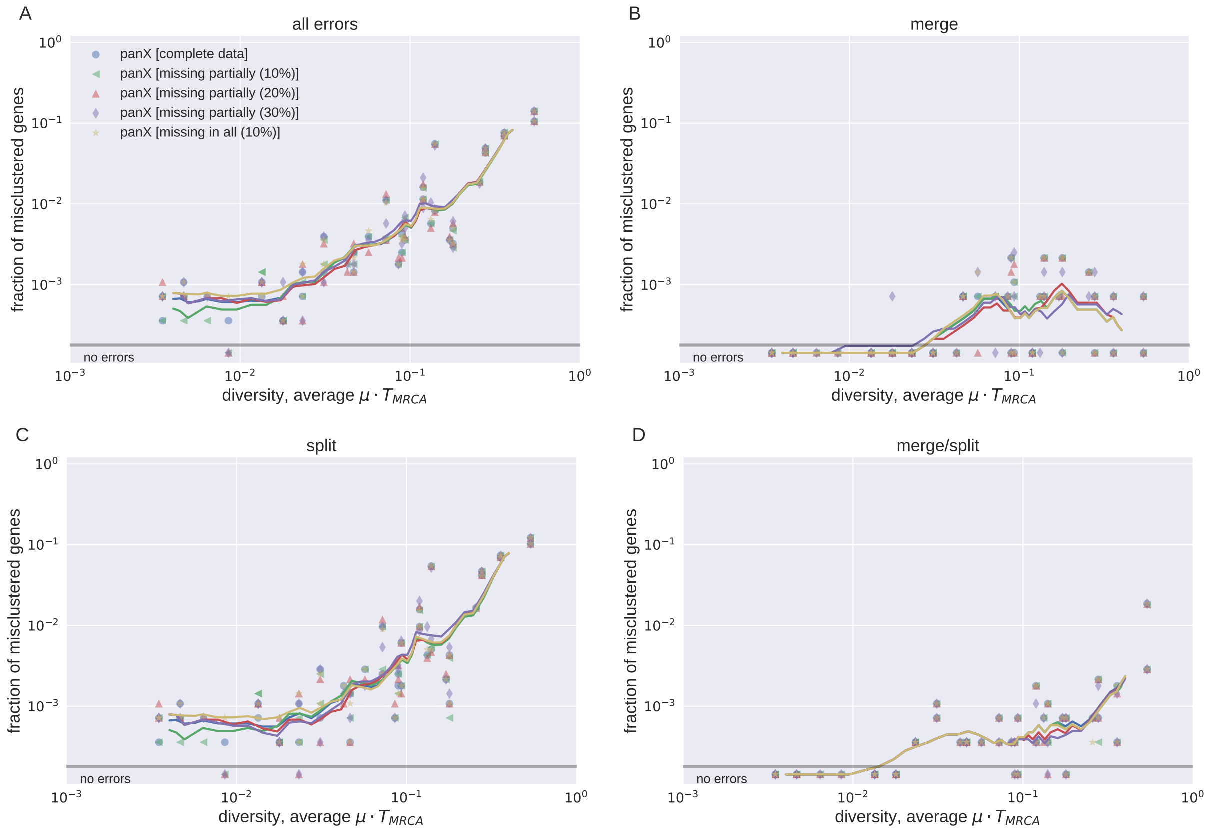

FIG. S5: Accuracy of clustering on different incomplete datasets. We created different simulation datasets with 10%, 20%, 30% of genes missing from 10%, 20%, 30% of strains (partial), respectively, and with 10% of genes missing in all strains. Panel A shows the sum of all clustering errors for different incomplete datasets as a function of the pan-genome diversity. Panels B-D show the fraction of clusters that contain additional genes (merge errors), incomplete clusters (split errors), and clusters that miss genes and contain extra genes (merge/split), respectively.

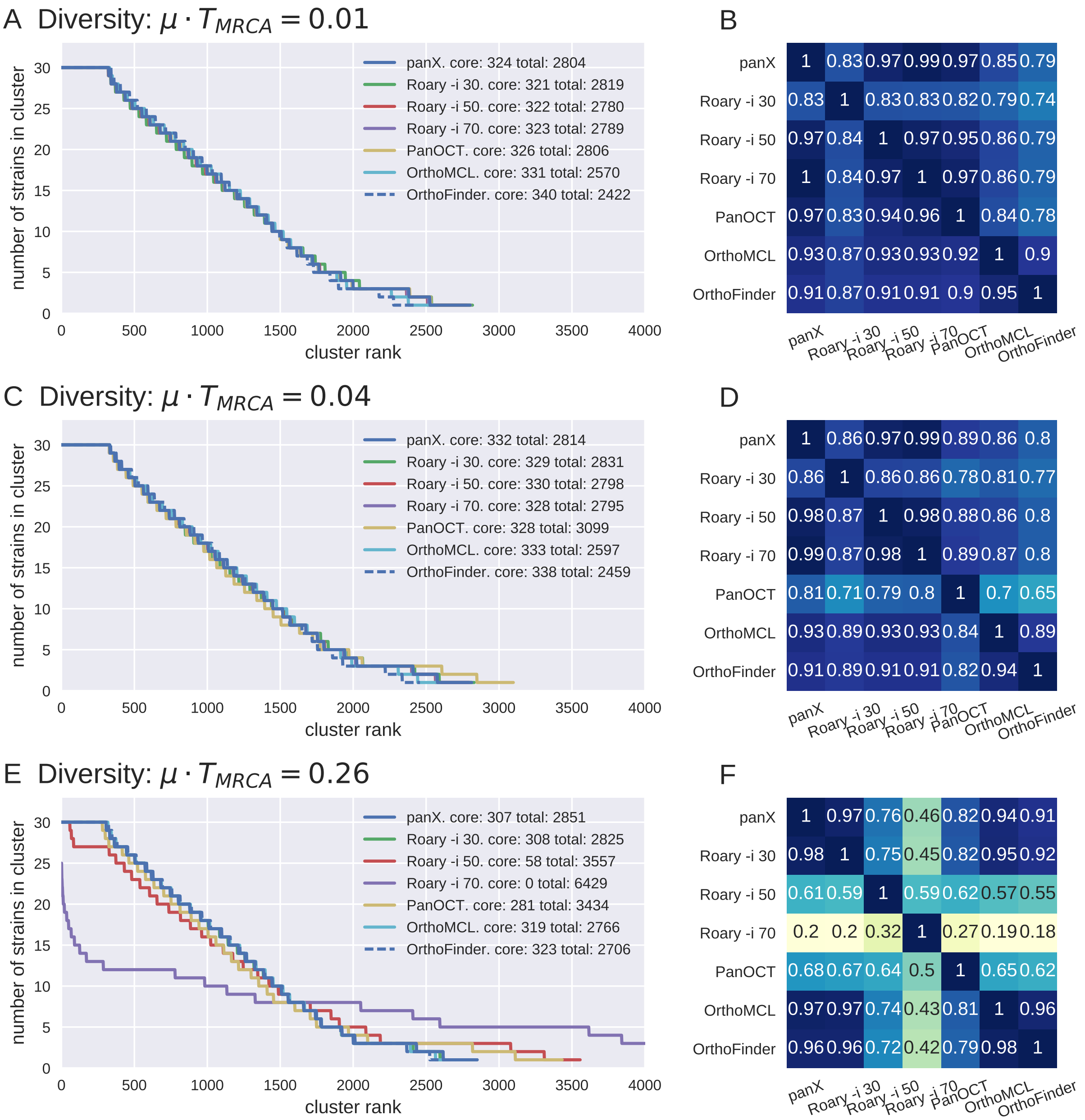

FIG. S6: Comparison of pan-genome inference from simulated genomes. Panel A, C & E show the size inverse cumulative cluster size distribution for simulated data sets of different diversities. All tools infer similar distributions for low diversity, where as several tools estimate fewer core genes and more small gene clusters at high diversity (gene clusters are identical, only the degree to which members of gene clusters mutated is different between panels). The right panels B, D & F show the fraction of clusters also found by other tools.
